# Development and Clinical Validation of a Finite Element Method Model Mapping Focal Intracranial Cooling

**DOI:** 10.1109/TNSRE.2022.3161085

**Published:** 2022-08-11

**Authors:** Turner S. Baker, Adantchede L. Zannou, Danna Cruz, Niranjan Khadka, Christopher Kellner, Richard Tyc, Marom Bikson, Anthony Costa

**Affiliations:** Sinai BioDesign, Icahn School of Medicine at Mount Sinai, New York, NY 10031 USA; Department of Neurosurgery, Icahn School of Medicine at Mount Sinai, New York, NY 10031 USA; Department of Biomedical Engineering, The City College of New York, CUNY, New York, NY 10031 USA.; Sinai BioDesign, Icahn School of Medicine at Mount Sinai, New York, NY 10031 USA.; Department of Biomedical Engineering, The City College of New York, CUNY, New York, NY 10031 USA; Department of Psychiatry, Harvard Medical School and Massachusetts General Hospital, Boston, MA 02115 USA.; Department of Neurosurgery, Icahn School of Medicine at Mount Sinai, New York, NY 10031 USA.; Monteris Medical Inc., Winnipeg, MB R3Y 1G4, Canada.; Department of Biomedical Engineering, The City College of New York, CUNY, New York, NY 10031 USA.; Sinai BioDesign, Icahn School of Medicine at Mount Sinai, New York, NY 10031 USA; Department of Neurosurgery, Icahn School of Medicine at Mount Sinai, New York, NY 10031 USA

**Keywords:** Therapeutic hypothermia, targeted temperature management, TTM, focal hypothermia, intracerebral hemorrhage, finite element methods, intracranial thermodynamics

## Abstract

Therapeutic hypothermia (TH) is a common and effective technique to reduce inflammation and induce neuroprotection across a variety of diseases. Focal TH of the brain can avoid the side effects of systemic cooling. The degree and extent of focal TH are a function of cooling probe design and local brain thermoregulation processes. To refine focal TH probe design, with application-specific optimization, we develop precise computational models of brain thermodynamics under intense local cooling. Here, we present a novel multiphysics in silico model that can accurately predict brain response to focal cooling. The model was parameterized from previously described values of metabolic activity, thermal conductivity, and temperature-dependent cerebral perfusion. The model was validated experimentally using data from clinical cases where local cooling was induced intracranially and brain temperatures monitored in real-time with MR thermometry. The validated model was then used to identify optimal design probe parameters to maximize volumetric TH, including considering three stratifications of cooling (mild, moderate, and profound) to produce Volume of Tissue Cooled (VOTC) maps. We report cooling radius increases in a nearly linear fashion with probe length and decreasing probe surface temperature.

## I. Introduction

THERAPEUTIC hypothermia (TH), or targeted temperature management (TTM), has been previously described as an effective neuroprotectant in laboratory and clinical studies by mitigating the effects of cerebral injury through a variety of mechanisms [1]–[3]. TTM provides several benefits for neurocompromised patients including the reduction of inflammation and limiting the negative effects of hypoxia [4], [5]. Systemic hypothermia, where body temperature is decreased throughout the body, has historically been the primary mechanism of TTM delivery [6]. Most often this approach is achieved with skin contact using cooling blankets [7], endovascular infusion of cold saline [8], or endovascular fluidics within closed-loop devices [9]. While this approach has proven successful in certain populations [10]–[14], many trials have been unable to demonstrate significant improvement in terms of clinical functional outcomes due to concurrent side effects associated with systemic cooling [15]. Severe shivering, pneumonia, cardiac arrhythmia, thrombocytopenia, and vascular dissection are associated with systemic hypothermia, which limits the potential of TTM-induced neuroprotection [16]–[19]. Many of these adverse effects can be minimized with the use of selective brain cooling, which reduces the burden on non-target organs while maximizing time-to-cooling at the primary site of injury.

The clinical practice of selective cerebral hypothermia dates back to 1965, however, effectively cooling deep brain structures via skin-surface cooling methods remains challenging, as sufficient heat flux is impossible transcranially [20]–[22]. Inducing TH intracranially may provide for rapid and effective cooling at the primary brain target site (e.g., region of compromised neurons) without the side effects associated with systemic cooling. Reductions in neuroinflammation and long-lasting neuroprotection have also been demonstrated in models of acute ischemic stroke and intracerebral hemorrhage (ICH) [23], [24].

Advancing technology for intracranial cooling is an important step toward developing targeted acute neuroprotective treatments. To optimize the development of focal cerebral hypothermia tools, numerical models of heat transfer between the probe and brain are critical. These models have integrated various intracranial regulation mechanisms including tissue-specific metabolic heat production, perfusion rate, blood temperature, and thermal conductivity [25]–[28]. Blood vessels form a dense network in which blood perfusion plays a central role in intracerebral thermoregulation [26], [29], [30]. Under normal conditions, internal brain temperature is tightly controlled by endogenous mechanisms to 37 °C. We have previously modeled incremental (∼1°C) heating around a neuromodulation implant [31]–[34], however, substantial cooling produces unique challenges to endogenous temperature regulation mechanisms, not adequately addressed in our previous modeling efforts.

Prior computational modeling has estimated the effects of focal cooling intracranially [35]–[38], predominantly in the field of ischemic stroke, but these studies lacked validation against clinical data. Some heating-based ablation probes use local cooling to regulate the spread of heat intracranially, and further monitor the regional brain temperature with magnetic resonance thermometry (MRT). Here, we present the first clinically validated in silico model that accurately predicts the brain’s response to focal cerebral cooling, informed by clinical cases of focal cooling monitored via MRT. Using this model, we demonstrate the importance of bioheat model parametrization including temperature-dependent cerebral perfusion and show how the design of focal cooling probes can shape the degree and region of cooling.

## II. Materials I& Methods

### A. Intraoperative Cooling and MR-Thermometry

Empirical MRT data was collected using the NeuroBlate System (Monteris Medical, USA). The NeuroBlate System is an MRI-guided laser ablation system that first induces focal, distributed during the laser interstitial thermal therapy (LITT). The cooling probe is internally cooled via CO2 gas expansion. The internal chamber of the 10 cm long, 3.3 mm diameter probe is cooled to a range of 1–4 °C, with the outer diameter reaching a temperature of approximately 15 °C. Brain temperature was recorded live through real-time MR thermometry using a fast radiofrequency-spoiled gradient recalled echo sequence (FOV 25.6 × 25.6 cm; matrix 128 × 128; TE 19.1 msec; TR 81 msec; flip angle 30°; bandwidth 100 Hz/pixel; and 3 slices of 5-mm thickness). This sequence required approximately 7.8 seconds for a single acquisition, and multiple acquisitions were obtained continuously. Thermometry data was calculated for each 2 × 2×5 mm voxel. Central voxels nearest the probe record the internal temperature of the probe and are sensitive to magnetic susceptibility artifacts, making many of the thermometry results within a 3 mm radius of the probe unreliable.

Postprocessing of the data was done in Python using the PyData ecosystem of libraries (numpy, pandas, SciPy, and skimage). Temperature values were processed as 2D images from each axial slice, and each patient and time point image was post-processed identically. Images were initially filtered with a multidimensional Gaussian filter (*σ* = 0.8) followed by an order 3 spline interpolation to a final voxel size of 0.5 × 0. 5 × 5.0 mm. The centroid of the cooling, and therefore the position of the probe, was determined visually. A simple distance matrix was used in cases where data is reported as a function of distance from the probe, with each voxel assigned a distance computed between the cell centroid and the point of probe origin.

### B. Model Setup and Parameters

**Fig. 1.**
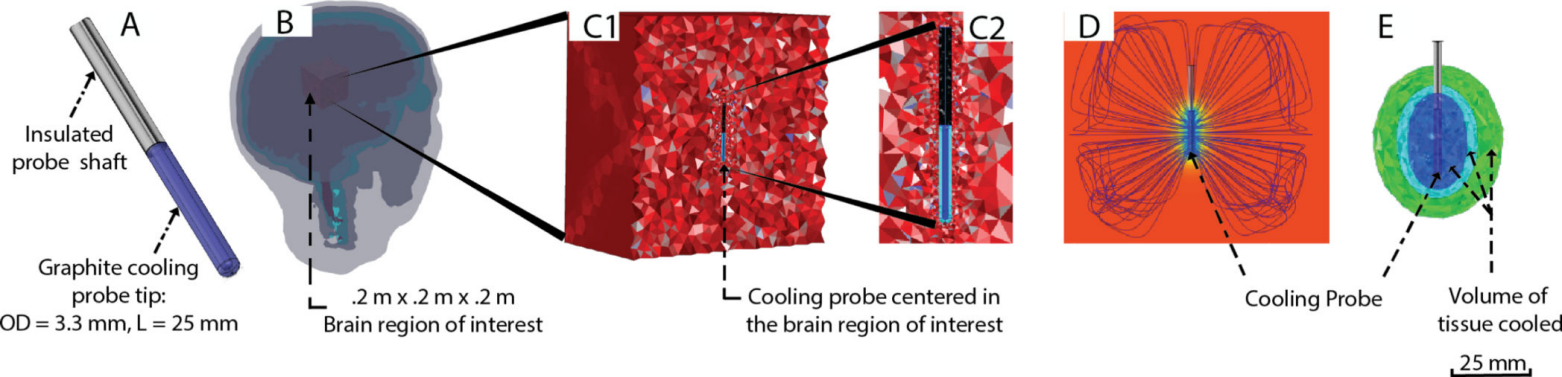
Time-dependent finite element method (FEM) bio-heat model dynamically predicts focal brain cooling during intracranial cooling. (A) A CAD-Derived model of the Monteris cooling probe. The probe was modeled as a 10 cm long, 3.3 mm diameter probe a temperature of approximately 15 °C, mirroring external diameter temperatures observed in benchtop and clinical results. (B) An MRI-derived segmented head model with positioned cooling probe. (C1) Cut view of a selected 3D region of interest (ROI) in the brain volume. (C2) Inset view of the meshed brain volume with the cooling probe. (D) Bio-heat model predicted focal brain cooling (false color) with the heat flux streamlines representing the spatial profile. (E) Associated representations of volume of tissue cooled (VOTC) of graded hypothermia volumes around the cooling probe: mild cooling (green), moderate cooling (light blue), and profound cooling (dark blue).

A magnetic resonance image-derived human head model was developed and segmented into various tissues including scalp, skull, cerebrospinal fluid (CSF), and brain using a series of automatic and manual segmentation algorithms in Simpleware (Synopsys, MA, USA). A computer-aided design (CAD)-derived Monteris cooling probe (OD 3.3 mm; L 25 mm, 12.5 mm, 50 mm) was modeled in SolidWorks 2016 (Dassault Systemes Americas Corp, MA, USA). An 8 × 106 mm3 region of interest (ROI) was selected inside the developed head model and simulated as a homogenous brain volume. The Monteris cooling probe was then positioned at the center of a selected ROI (Figure 1B). The model assembly was imported into COMSOL Multiphysics 5.1 (COMSOL Inc., MA, USA), and meshed using a built-in voxel-based meshing algorithm (Figure 1C). The geometry model was comprehensively discretized into 166,031 tetrahedral elements (element quality = 0.75; element volume ratio = 2.72 × 10–6; mesh volume = 0.008 m3), 8,992 triangular elements (element quality = 0.98; element area ratio = 1.59 × 10–4; mesh face area = 0.241 m2), 606 edge elements (element length ratio = 0.019; mesh edge length = 2.69 m; growth rate = 1.68), and 22 vertex elements. The subsequent meshes were computationally solved using the Finite Element Method (FEM).

The standard Pennes’ bio-heat transfer equation was implemented and solved using a local temperature dependent adaptive function in time domain as described below:

(1) $$\frac{\rho C_{p}\delta T}{\delta t}=-\nabla (\kappa \nabla T)+\rho_{b}C_{b}\omega_{b}\left(T-T_{b}\right)+Q_{v}+Q_{met}$$

where, Cp, T„ b, Qv, and Qmet respectively represent tissue density, specific heat, temperature, thermal conductivity, blood perfusion rate, volumetric cooling source, and metabolic heat generation rate in the brain.

Several references on human biology, physiology, and histology were reviewed providing an approximate value for each of the parameters under normal conditions: Brain tissue was modeled with a specific heat capacity of 3250J/(kg·K), density of 1040kg/m3, and thermal conductivity of 0.51W/(m· K) [39], [40]. Similarly, in-vivo studies were cross-referenced to adapt the brain blood perfusion rate, b, and the brain tissue metabolic rate, Qmet, as functions of the local brain tissue temperature, T, as described by equation (2) [35], [41]–[43]. An exponential relationship was established between the local brain tissue temperature and the brain blood perfusion rate and brain metabolic rate:

$$\begin{array}{c} \omega_{b}(\text{T})=0.0012\text{e}0.054\text{T and }Q_{met}(\text{T})=342.45\text{e}0.091\text{T}\\ \frac{\rho C_{p}\delta T}{\delta t}=-\nabla (\kappa \nabla T)+\rho_{b}C_{b}\omega_{bEff}\left(T-T_{b}\right)+Q_{b}+Q_{met}Eff \end{array}$$

wb-Eff and Qmet-Eff respectively represent the effective blood perfusion rate and the effective tissue metabolic heat generation rate in the brain as described in equations (3) and (4).


(3) $$\omega_{bEff}=\omega_{b}(T)$$

(4) $$Q_{met}Eff=Q_{met}(T)$$

Other physical properties such as specific heat, Cp, temperature, Tb, and density, *ρ*b, were assumed constant in the brain tissues [37], [44]. Note that the arterial blood temperature was modeled at 0.3 °C less than the core temperature (37 °C) to account for the convective thermal energy transport throughout the brain through vascularization [45]–[47].

The outer shell of the probe tip was modeled as sapphire with thermal conductivities (), heat capacity (Cp), and density () set as 25.2 W/(m K), 757.304 J/(kg K), 3980 kg/m3, respectively. The core of the cooling probe was modeled as CO2. while the outer shell was simulated as graphite The result is an outer probe surface temperature of approximately 15 °C as experimentally obtained. The probe surface temperature was also varied from 0 °C to 36 °C for probe optimization purposes. The outer boundary of the brain volume was fixed at 37 °C. Convective heat loss to the ambient environment and convective gradients across brain tissues were assumed nonexistent [37], [48]–[50]. The model, 8 × 106 mm3 brain volume was confirmed sufficiently large to reasonably represent the entire brain.

### C. Volume of Tissue Cooled (VOTC)

The VOTCs were computed using volumetric integration with the threshold temperatures set to 36 °C, 34 °C, and 32 °C respectively for mild hypothermia, moderate hypothermia, and profound hypothermia. The area cooled was estimated radially at mid-height and along the axial plane of the cooling probe tip.

### D. Statistical Analysis

The accuracy of the computational model was determined through a comparison of the radial cooling and the area of TH over time. Temperature data at each voxel that intersected concentric circles of specified radial distances were collected at each time point. Standard errors were calculated for the dataset of each radial distance. The area of clinically relevant cooling was calculated between two separate cooling datasets, and the mean and standard error were calculated for the cooling times available. Graphics were created using GraphPad Prism (Version 9.0.0. GraphPad Software, San Diego, CA, USA).

## III. Results

### A. Focal Response to Direct Intracranial Cooling

**Fig. 2.**
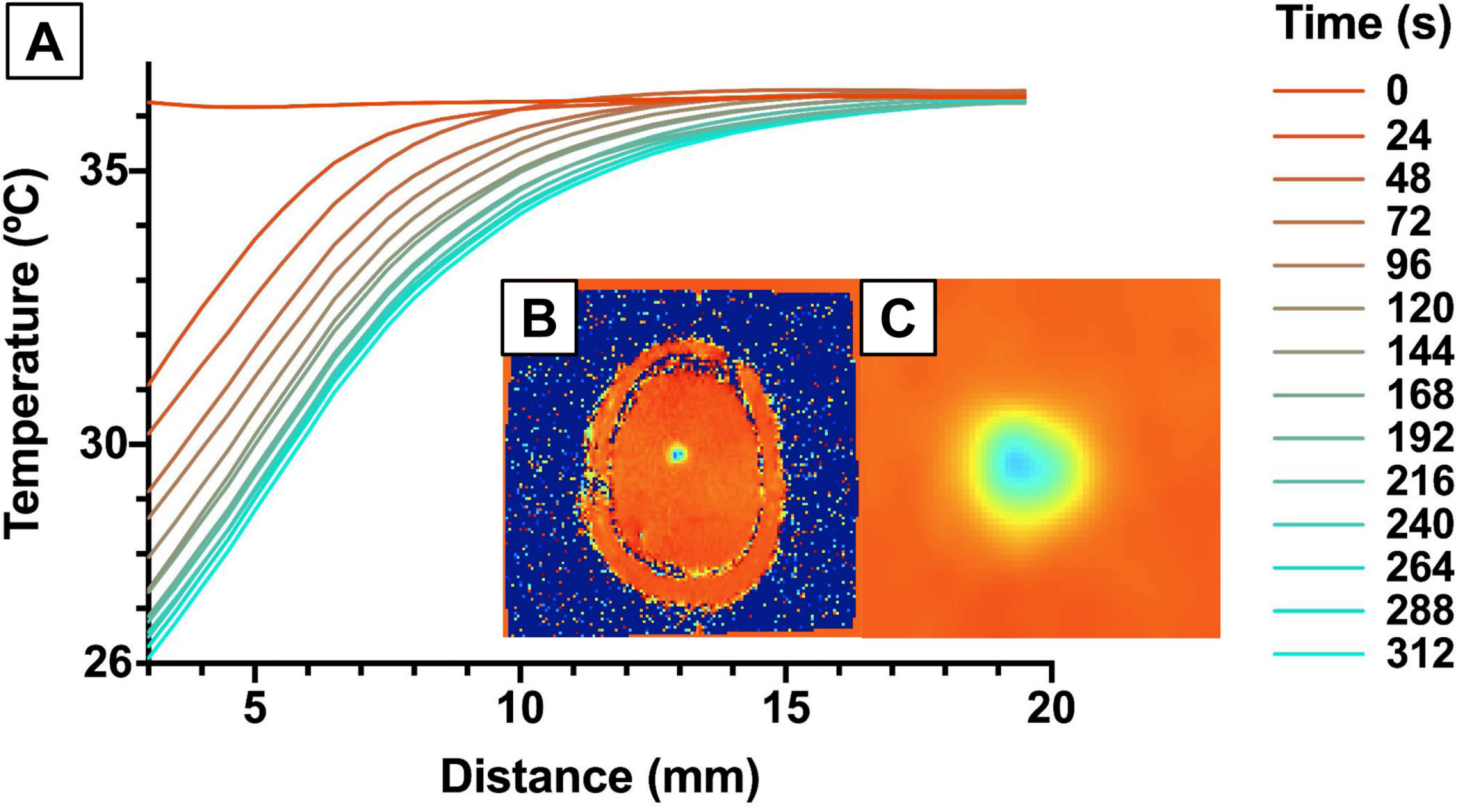
Clinical effect of focal intracranial cooling. (A) Temperature as a function of distance from a cooling probe initiated at 15 °C in a clinical setting. Mean temperature is displayed from 3–20 mm radial distance from the probe over the full period of cooling (312 seconds). Significant cooling is rapidly induced within the 5 mm of the probe, within minimal effect beyond 13 mm. (B) Temperature profile at t = 296 s before data post-processing. (C) Temperature profile at t = 296 s after data postprocessing (see Methods, above).

Two clinical cases that had extended cooling periods prior to the initiation of tissue ablation were selected, lasting between 136–312 seconds. Imaged temperature changes in response to local cooling of the more prolonged cases are presented in Figure 2. Given the size of the sampling voxel in the original MRT analysis (2 mm) and susceptibility artifact (∼ 3 mm), we report temperature measurements at a radius of >3 mm. Cooling was found to decrease significantly at increased distances, with minimal cooling seen at a distance >13 mm from the probe. Brain tissue near the probe (< 5 mm) saw a rapid reduction in temperature following probe activation, decreasing below 33 °C in under 30 seconds.

### B. Computational Model Accurately Maps Intracranial Thermodynamic Response to Focal Cooling

**Fig. 3.**
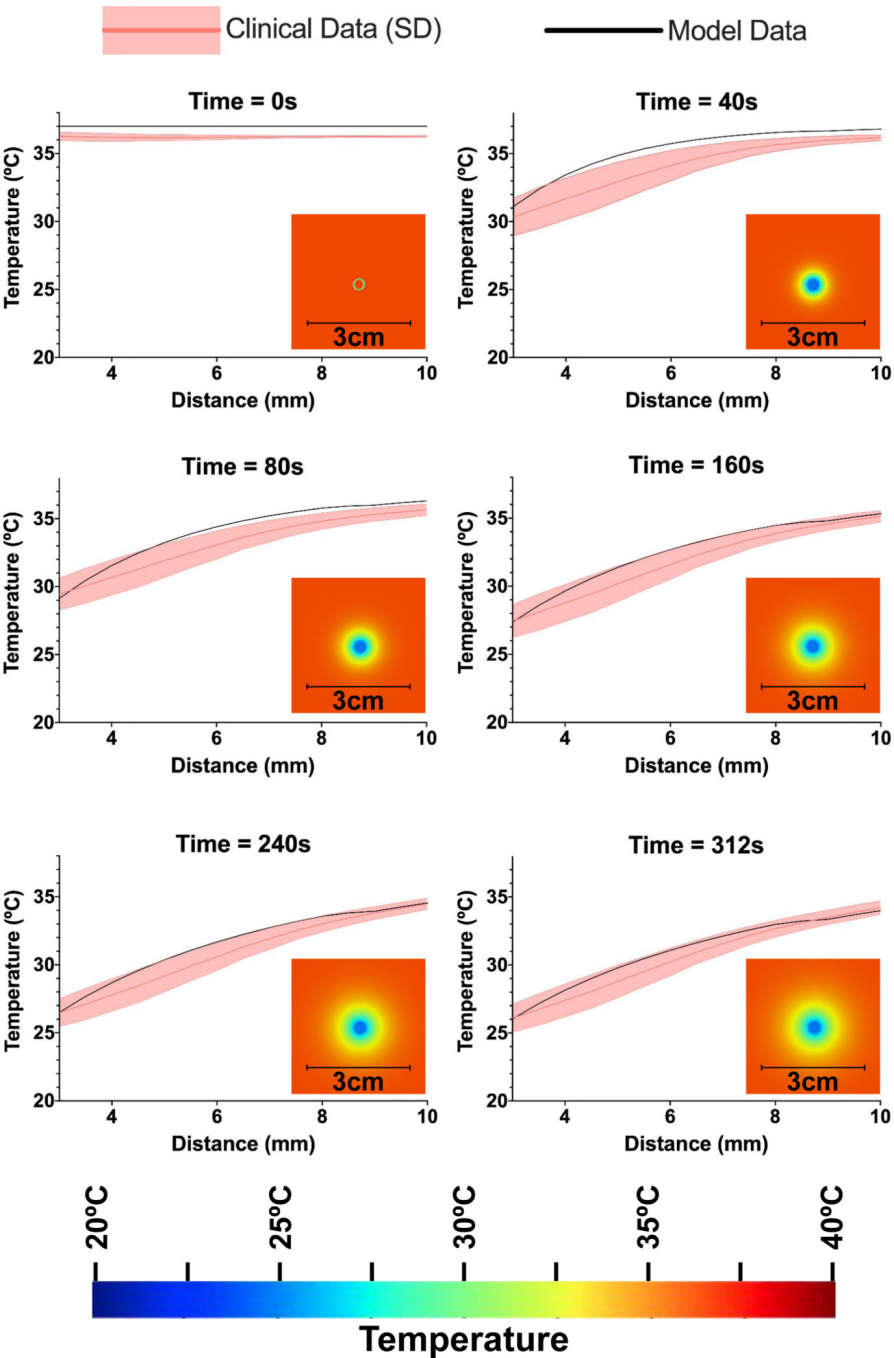
Comparing radial model cooling to clinical data. The average radial temperature of the clinical data is depicted (solid red) along with average radial error at each given distance (light red), compared to the computational model described across six time points. Visualization of radial cooling data within the model is shown at each time point. The computational model closely mirrors the clinical data, while slightly underestimating the effect of focal, intracranial cooling at early (<80 seconds) timepoints.

Computational model predictions of brain temperature are shown along with clinical data at time points distributed across the cooling period (Figure 3). Temperature is plotted as a function of distance from the probe center. The degree and spatial extent of cooling increased with time. Change in brain temperature was assumed to extend in a radially symmetric fashion, and variation in local temperature at specific distances was used to generate error bars for the clinical data. Only one of the available clinical datasets had prolonged cooling (>140 seconds), so errors were calculated from a single clinical case. The model’s predicted cooling was within the range expected from clinical MR thermometry observations. Moderate differences between simulated and measured data may reflect data offsets (e.g., evident at time 0 s).

### C. Utilizing Validated Thermodynamic Model to Optimize Cooling Probe Design

Having validated the computational model against the clinical data using a fixed probe design, we used the model to refine probe designs to maximize the induction of TH. Applied probe surface temperature (0–40 °C) and length (0.5x and 2x) were assessed (Figure 5) at a cooling time of one hour. The area (2D, axial slice at probe midpoint) and volume of clinically relevant cooling temperatures (for mild, moderate, and profound hypothermia stratification) were assessed. The surface temperature serves as a downstream multivariate surrogate for probe material selection, material thickness, and internal probe temperature. For each stratification, the area of hypothermia was found to decrease in a quasi-linear fashion in response to probe surface temperature (Figure 5A-C), as did the volume of hypothermia (Figure 5D-F). Halving probe length reduced mild cooling by a maximum area of 28% and a maximum volume of 48%. Doubling probe length increased the maximum area of mild cooling by 34 percent compared to the original length, and maximum volume by 99%.

**Fig. 4.**
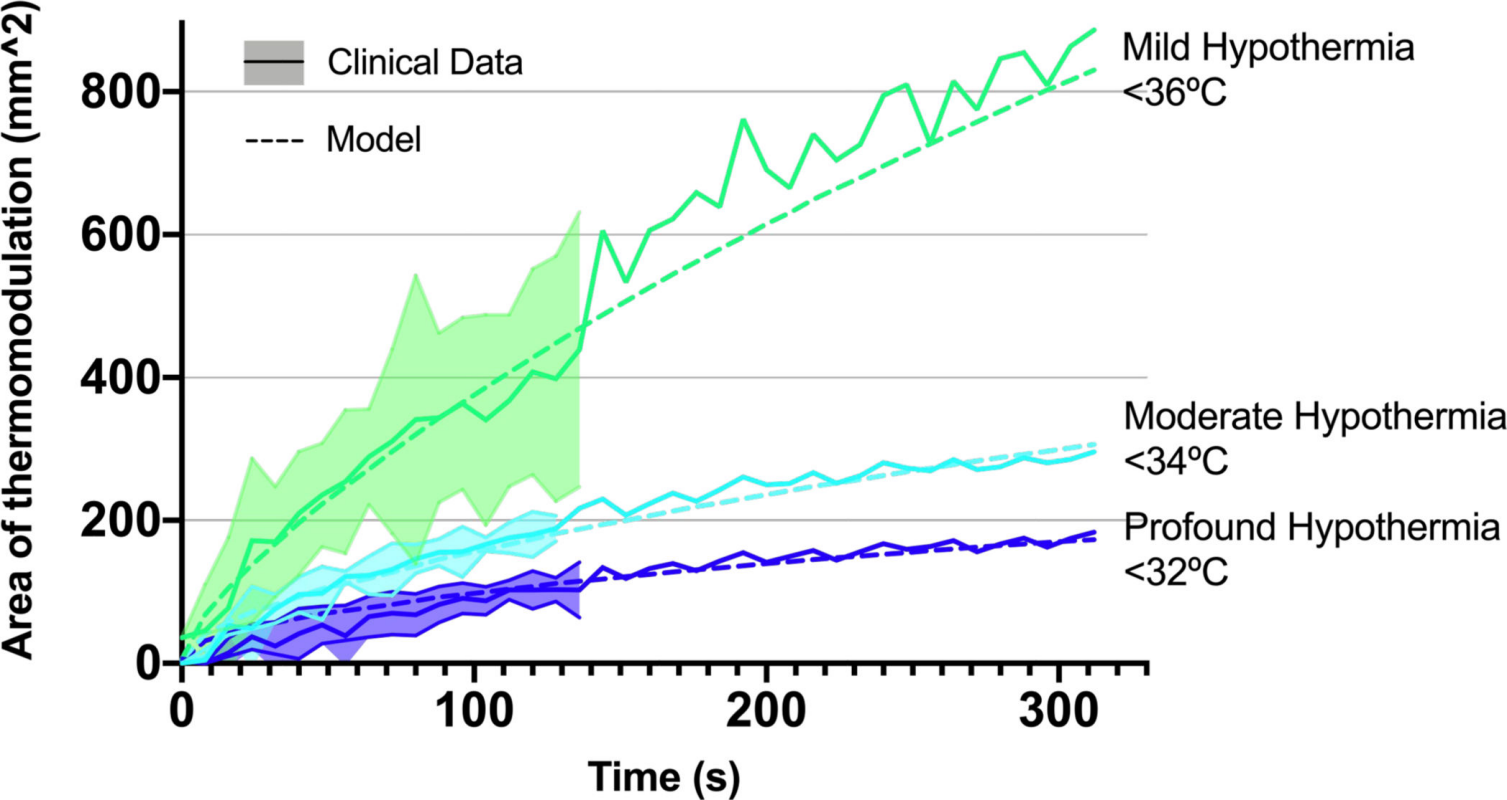
Clinically relevant cooling areas. Area of induced therapeutic hypothermia after 312 seconds of focal cooling for both the clinical (solid line) and model (dashed line) results. Clinical data represent a mean of two clinical cases with error indicated until the end of the shorter case at approximately 130 seconds. Area of mild (<36°C, green), moderate (<34°C, light blue), and profound (<32°C, dark blue) were assessed independently. Induction of mild does not appear to reach steady-state, while moderate and profound hypothermia levels appear to be asymptotically approaching a maximum.

**Fig. 5.**
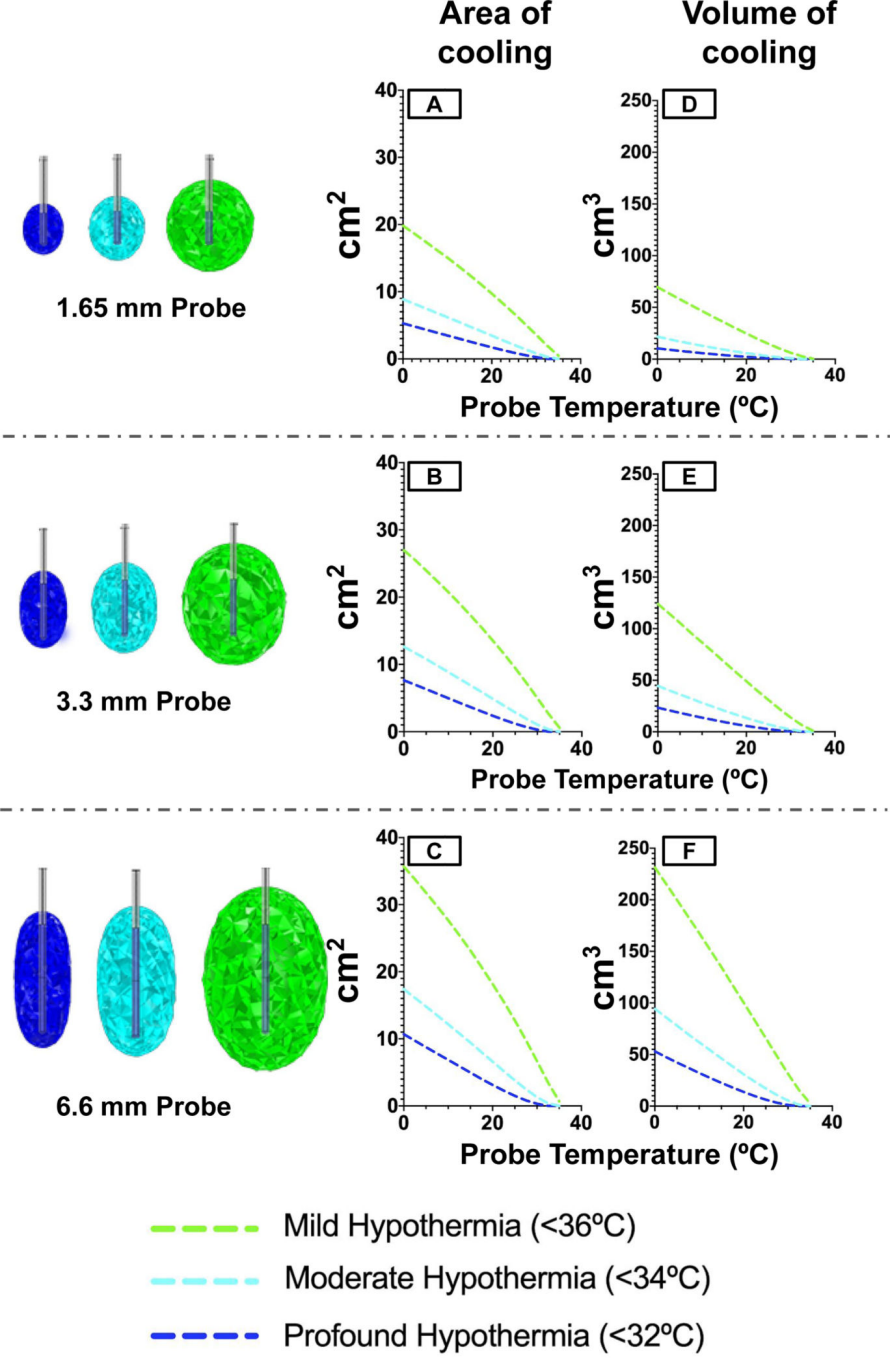
Volume of Tissue Cooled (VOTC) maps. The amount of tissue at specific TH levels is indicated as a function of temperature across three probe lengths in both the area around the center point of the probe (A-C) and total volume of the 3D model (D-F). The amount of tissue that reached mild hypothermia (<36°C, green), moderate hypothermia (<34°C), and profound hypothermia (<32°C) was assessed. Standard probe lengths (A,D) were compared to probes that were 2X in length (B,D) and 0.5X (C,F). A visual representation of volumetric cooling was generated for each of the varied probe lengths.

The area of cooling recorded by MR thermometry was compared with computational model predictions over time, computed using three common stratifications of cooling in TH: mild (<36 °C), moderate (<34 °C), and profound (<32 °C) (Figure 4). Two independent clinical cases were included with mean and standard error of the area of thermomodulation calculated as a function of time. = Areas of cooling increased with time for all stratifications. As expected, the area of cooling increases with decreasing cooling threshold (mild > moderate > profound). The model fell within the inter-patient variability across all stratifications up to 130 seconds, and tracked closely with the single clinical case that induced prolonged cooling up to 312 seconds.

The extent and specificity of cooling can be controlled by probe design. For example, achieving a target mild hypothermic tissue volume of 100 cm3 after one hour of cooling would require a standard probe to operate at a surface temperature of 6.3 °C (with a corresponding profound hypothermic tissue volume of 16.18 cm3), a 2X length probe to operate at a surface temperature of 20 °C (with a corresponding profound hypothermic tissue volume of 16.94 cm3), and would not be possible to achieve with a 0.5X length probe even at 0 °C.

## IV. Discussion

Clinically, focal brain cooling was found to induce clinically-relevant temperature reductions at relatively small distances from the probe. Minimal cooling was observed beyond 13 mm, and the maximum, stable temperature reduction measurement (at 3 mm from the probe surface) was approximately 9 °C. This small radius of cooling is potentially explained by the high arterial perfusion rate within the brain’s core, acting to minimize the thermodynamic gradient intracranially. Brain perfusion rate has been shown to decrease as a function of the surrounding tissue temperature, but may only enact change on the local temperature at a specific threshold, explaining the steep change in temperature seen approximately 7 mm from the probe (Figure 2). While the internal probe temperature of the cooling probe was set to 0 °C, the sapphire tip reduces the efficacy of heat transfer, resulting in a predicted probe surface temperature of 15 °C.

This modest radial cooling and steep temperature gradient is also suggestive that transcranial or transnasal approaches to induce TH in regions of the brain greater than 5 mm from the point of cooling will have limited efficacy. This is especially true for transcranial techniques, where the skull and scalp act as an additional insulating barrier.

### A. Accuracy and Generalization of the Computational Model

The developed model successfully reproduced clinically-observed time-dependent temperature distributions following local induction of direct tissue cooling. The model accurately recreated the intracranial thermodynamic response to focal cooling, showing a mild cooling area of 363 mm2 at 136 seconds, well within the error range of the two clinical cases assessed. Inter-patient variability of thermodynamic responses was low, but future studies would still benefit from additional assessments to test for the generalizability of the findings across a larger cohort. A prospective study ensuring elongated cooling prior to ablation would also benefit the refinement of the described model. Brain anatomy was not taken into account in this model, and vasculature, brain regions, and proximity to the core or scalp likely play an important role in the thermodynamic characteristics of the tissue.

### B. Potential of the Computational Model

The computational model was demonstrated to be a useful tool in optimizing the design of internal cooling probes, and future studies further refining an optimal intracranial cooling element design are underway. Probe surface temperature was found to be the most crucial design element affecting the area of therapeutic cooling. This is consistent with the hypothesis that the minimal radial cooling seen in the clinical data is due to the use of the insulating sapphire probe material, which limits temperature at the probe surface. Most systemic TH devices use thermally conductive materials, such as PEBAX, to maximize heat transfer intravenously. Probe length is also crucial in controlling the extent of cooling in total volume and maximum distance. While ideal intracranial probes will minimize brain tissue displacement, probe length will be an important factor in selecting cooling probe designs to maximize cooling effect. The potential benefits of an accurate and multiphysics 3D thermodynamic brain model, validated by clinical data, extend beyond focal hypothermia applications. These models can be adapted to support medical device designs and therapy optimization within the fields of tissue ablation, electrode deployment and activation, and antipyretic therapies.

### C. Limitations of the Described Study

There are numerous limitations to the described experimental study. Only two clinical cases with significant cooling periods were identified, and a future study containing a larger volume of clinical cases would vastly improve the reliability of the model’s accuracy. The probe was modeled with an external temperature of 15°C, which was provided by the manufacturer but may vary slightly from the actual external temperature of the probe within each clinical setting. The in-silico brain was modeled as a single body, and did not contain sulci or ventricles, which do not receive direct arterial blood flow; this may lead to the model underestimating the radial cooling. The brain model also modeled arterial blood temperature as a constant, which fails to account for the potential of slight systemic cooling as a result of localized cerebral cooling. The location of the probe within each clinical case was not included in the model, and may affect the VOTC, particularly if near sulci or ventricles.

## V. Conclusion

This study utilized a novel multiphysics in silico model of the brain’s thermodynamic response to localized cooling. This is the only identified intracranial thermodynamic to be validated directly against clinical data. Intracranial cooling was found to have a limited radius of effect, suggesting that intracranial cooling probes would need to be further optimized for use in therapeutic hypothermic interventions. The model was found to be functional in assessing probe designs, finding cooling radius to increase linearly with probe length and decrease linearly with probe surface temperature, and will benefit from further refinement through additional anatomical detail.
